# Crystal structure of 2-amino-4-(4-chloro­phen­yl)-1-(4-methyl­phen­yl)-5-oxo-1,4,5,6,7,8-hexa­hydro­quinoline-3-carbo­nitrile

**DOI:** 10.1107/S2056989015021313

**Published:** 2015-11-14

**Authors:** Shaaban K. Mohamed, Mehmet Akkurt, Jerry P. Jasinski, Omyma A. A. Abd Allah, Mustafa R. Albayati

**Affiliations:** a Chemistry and Environmental Division, Manchester Metropolitan University, Manchester M1 5GD, England; b Chemistry Department, Faculty of Science, Minia University, 61519 El-Minia, Egypt; c Department of Physics, Faculty of Sciences, Erciyes University, 38039 Kayseri, Turkey; d Department of Chemistry, Keene State College, 229 Main Street, Keene, NH 03435-2001, USA; e Chemistry Department, Faculty of Science, Sohag University, 82524 Sohag, Egypt; f Kirkuk University, College of Science, Department of Chemistry, Kirkuk, Iraq

**Keywords:** crystal structure, di­hydro­pyridines, annelated di­hydro­pyridines, hydrogen bonding, inversion dimers

## Abstract

In the title compound, C_23_H_20_ClN_3_O, each of the cyclo­hexene and 1,4-di­hydro­pyridine rings of the 1,4,5,6,7,8-hexa­hydro­quinoline ring system adopts a twisted-boat conformation. The dihedral angle between the two benzene rings is 11.52 (7)°. In the crystal, mol­ecules are linked through a pair of amino–nitrile N—H⋯N hydrogen bonds, forming inversion dimers. These assemble into a three-dimensional network *via* C—H⋯O and C—H⋯π inter­actions.

## Related literature   

For the synthesis and pharmaceutical applications of di­hydro­pyridines, see: Kumar & Maurya (2007 ▸); Kendre *et al.* (2008 ▸); Heydari *et al.* (2009 ▸).
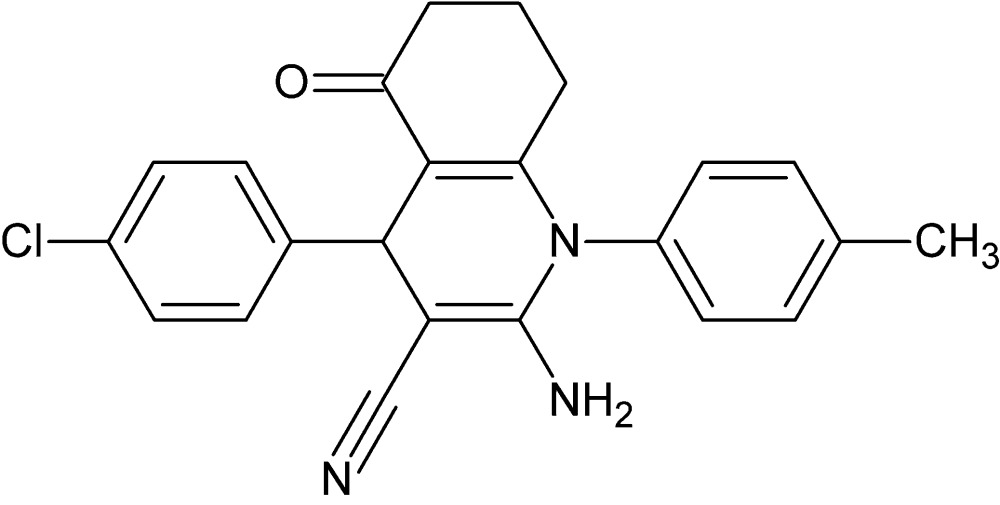


## Experimental   

### Crystal data  

C_23_H_20_ClN_3_O
*M* *_r_* = 389.87
Monoclinic, 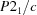

*a* = 8.7759 (3) Å
*b* = 10.6399 (3) Å
*c* = 20.7929 (7) Å
β = 93.842 (3)°
*V* = 1937.17 (11) Å^3^
*Z* = 4
Mo *K*α radiation
μ = 0.22 mm^−1^
*T* = 293 K
0.46 × 0.42 × 0.38 mm

### Data collection  

Agilent Xcalibur Eos Gemini diffractometer
Absorption correction: multi-scan (*CrysAlis PRO*; Agilent, 2014 ▸) *T*_min_ = 0.841, *T*_max_ = 1.000
15538 measured reflections
6453 independent reflections
4853 reflections with *I* > 2σ(*I*)
*R*_int_ = 0.027

### Refinement  

*R*[*F* ^2^ > 2σ(*F* ^2^)] = 0.053
*wR*(*F* ^2^) = 0.144
*S* = 1.04
6453 reflections
254 parameters
H-atom parameters constrained
Δρ_max_ = 0.50 e Å^−3^
Δρ_min_ = −0.48 e Å^−3^

### 

Data collection: *CrysAlis PRO* (Agilent, 2014 ▸); cell refinement: *CrysAlis PRO*; data reduction: *CrysAlis PRO*; program(s) used to solve structure: *SHELXS2014* (Sheldrick, 2008 ▸); program(s) used to refine structure: *SHELXL2014* (Sheldrick, 2015 ▸); molecular graphics: *ORTEP-3 for Windows* (Farrugia, 2012 ▸); software used to prepare material for publication: *PLATON* (Spek, 2009 ▸).

## Supplementary Material

Crystal structure: contains datablock(s) global, I. DOI: 10.1107/S2056989015021313/tk5407sup1.cif

Structure factors: contains datablock(s) I. DOI: 10.1107/S2056989015021313/tk5407Isup2.hkl

Supporting information file. DOI: 10.1107/S2056989015021313/tk5407Isup3.cml

**Fig. 1.**
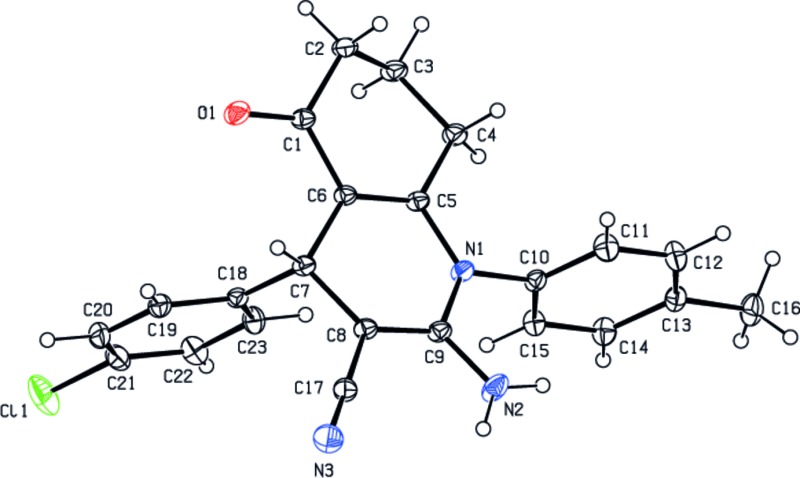
Molecular structure of the title compound with displacement ellipsoids drawn at the 30% probability level.

**Fig. 2.**
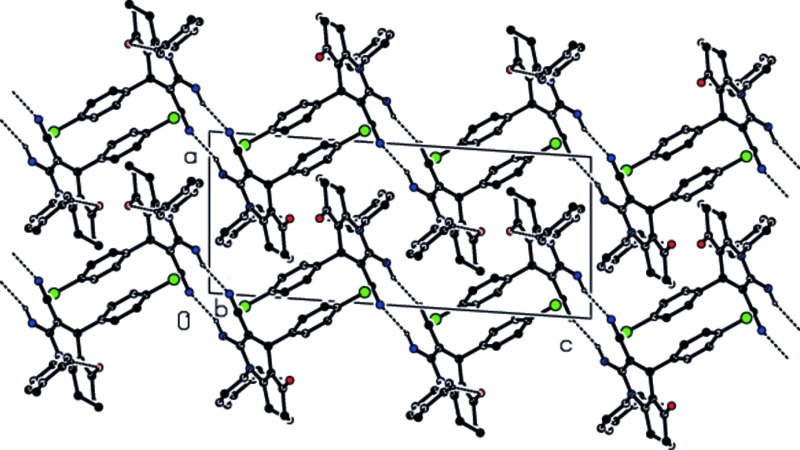
View of the dimers formed by N—H···N hydrogen bonds down the b axis.

. DOI: 10.1107/S2056989015021313/tk5407fig1.tif
Mol­ecular structure of the title compound with displacement ellipsoids drawn at the 30% probability level.

b . DOI: 10.1107/S2056989015021313/tk5407fig2.tif
View of the dimers formed by N—H⋯N hydrogen bonds down the *b* axis.

CCDC reference: 1436038

Additional supporting information:  crystallographic information; 3D view; checkCIF report

## Figures and Tables

**Table 1:** Hydrogen-bond geometry (Å, °) *Cg*3 and *Cg*4 are the centroids of the methyl- and chloro-benzene rings (C10–C15 and C18–C23), respectively.

*D*—H⋯*A*	*D*—H	H⋯*A*	*D*⋯*A*	*D*—H⋯*A*
N2—H2*N*⋯N3^i^	0.91	2.17	3.013 (2)	153
C14—H14⋯O1^ii^	0.93	2.58	3.4577 (19)	156
C3—H3*A*⋯*Cg*4^iii^	0.97	2.93	3.7210 (16)	139
C16—H16*B*⋯*Cg*3^iv^	0.96	2.81	3.6464 (18)	146

Symmetry codes: (i) 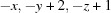; (ii) 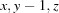; (iii) 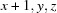; (iv) 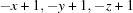.

## supplementary crystallographic information

### S1. Comment

Dihydropiridines (DHP's) are a significant class of heterocyclic molecules due to their important biological activities (Kumar & Maurya, 2007). Dihydropyridine drugs, such as nifedipine, nicardipine and amlodipine are well known as cardiovascular agents for the treatment of hypertension (Kendre *et al*., 2008), calcium channel modulators and for cardiovascular disease treatment (Heydari *et al.*, 2009). In this context, we report here the synthesis and crystal structure of the title compound.

In the title compound (Fig. 1), the cyclohexene (C1–C6) and 1,4-dihydropyridine (N1/C5–C9) rings of the 1,4,5,6,7,8-hexahydroquinoline ring system (N1/C1–C9) each adopt a twisted-boat conformation (the puckering parameters are Q_T_ = 0.4813 (16) Å, θ = 123.31 (18)°, φ = 283.9 (2)° and Q_T_ = 0.2182 (14) Å, θ = 103.7 (4)°, φ = 353.5 (4)°, respectively). The dihedral angle between the methyl- and chloro-benzene rings is 11.52 (7)°.

In the crystal, a pair of N—H···N intermolecular hydrogen bonds connects two molecules by an *R*_2_^2^(12) ring motif (Table 1), forming a centrosymmetric dimer (Fig. 2). Weak C—H···O and C—H···π interactions connect the dimers to each other, forming a three-dimensional network.

### S2. Experimental

To a solution of 1.3-cyclohexanedione (3.36 g, 0.03 mol) in 40 ml ethanol, *p*-toluidine (3.21 g, 0.03 mol) and catalytic a amount of triethylamine were added. The mixture was heated under reflux for 3 h. (4-Chlorobenzylidene)malononitrile (5.68 g, 0.03 mol) was added to the reaction mixture and refluxed for another 3 h. The separated solid was filtered off while hot, dried and crystallized from DMF as colourless crystals. Yield: 49.7%; m.p. 573 K, IR (λ_max_, cm^-1^): 3472, 3325 (NH_2_), 3209, 3032 (CH_arom._), 2972–2879 (CH_aliph._), 2177 (C≡N), 1631 (C=O); ^1^H-NMR (DMSO-d_6_), δ ppm: 7.39–7.29 (m, 8H, CH_arom._), 5.32 (s, 2H, NH_2_, masked by D_2_O), 4.51 (s, 1H, CH), 2.4 (s, 3H, CH_3_), 2.23–2.19 (t, 2H, CH_2_—C=O), 1.93–1.81 (t, 2H, CH_2_—C=C), 1.67–1.62 (m, 2H, CH_2_-CH_2_-CH_2_); ^13^C-NMR (DMSO-d_6_), δ ppm: 195.45, 153.14, 151.70, 146.07, 139.86, 133.96, 131.26, 131.03, 130.10, 129.13, 128.79, 121.78, 112.72, 60.26, 36.45, 36.21, 28.23, 21.22, 21.06.

### S3. Refinement

All H atoms were placed in calculated positions with N2—H1N = 0.90 Å, N2—H2N = 0.91 Å, and C—H = 0.93 - 1.04 Å, and refined as riding with *U*_iso_(H) = 1.2 or 1.5*U*_eq_(C, N).

### Crystal data

**Table:**

C_23_H_20_ClN_3_O	*F*(000) = 816
*M**_r_* = 389.87	*D*_x_ = 1.337 Mg m^−^^3^
Monoclinic, *P*2_1_/*c*	Mo *K*α radiation, λ = 0.71073 Å
Hall symbol: -P 2ybc	Cell parameters from 4336 reflections
*a* = 8.7759 (3) Å	θ = 3.5–32.1°
*b* = 10.6399 (3) Å	µ = 0.22 mm^−^^1^
*c* = 20.7929 (7) Å	*T* = 293 K
β = 93.842 (3)°	Irregular, colourless
*V* = 1937.17 (11) Å^3^	0.46 × 0.42 × 0.38 mm
*Z* = 4	

### Data collection

**Table:**

Agilent Xcalibur Eos Gemini diffractometer	6453 independent reflections
Radiation source: Enhance (Mo) X-ray Source	4853 reflections with *I* > 2σ(*I*)
Graphite monochromator	*R*_int_ = 0.027
Detector resolution: 16.0416 pixels mm^-1^	θ_max_ = 32.8°, θ_min_ = 3.5°
ω scans	*h* = −13→12
Absorption correction: multi-scan (*CrysAlis PRO*; Agilent, 2014)	*k* = −15→13
*T*_min_ = 0.841, *T*_max_ = 1.000	*l* = −31→29
15538 measured reflections	

### Refinement

**Table:**

Refinement on *F*^2^	0 restraints
Least-squares matrix: full	Hydrogen site location: mixed
*R*[*F*^2^ > 2σ(*F*^2^)] = 0.053	H-atom parameters constrained
*wR*(*F*^2^) = 0.144	*w* = 1/[σ^2^(*F*_o_^2^) + (0.0563*P*)^2^ + 0.8789*P*] where *P* = (*F*_o_^2^ + 2*F*_c_^2^)/3
*S* = 1.04	(Δ/σ)_max_ < 0.001
6453 reflections	Δρ_max_ = 0.50 e Å^−^^3^
254 parameters	Δρ_min_ = −0.48 e Å^−^^3^

### Special details

**Table:**

Geometry. Bond distances, angles *etc*. have been calculated using the rounded fractional coordinates. All su's are estimated from the variances of the (full) variance-covariance matrix. The cell e.s.d.'s are taken into account in the estimation of distances, angles and torsion angles
Refinement. Refinement on *F*^2^ for ALL reflections except those flagged by the user for potential systematic errors. Weighted *R*-factors *wR* and all goodnesses of fit *S* are based on *F*^2^, conventional *R*-factors *R* are based on *F*, with *F* set to zero for negative *F*^2^. The observed criterion of *F*^2^ > σ(*F*^2^) is used only for calculating -*R*-factor-obs *etc*. and is not relevant to the choice of reflections for refinement. *R*-factors based on *F*^2^ are statistically about twice as large as those based on *F*, and *R*-factors based on ALL data will be even larger.

### Fractional atomic coordinates and isotropic or equivalent isotropic displacement parameters (Å^2^)

**Table:**

	*x*	*y*	*z*	*U*_iso_*/*U*_eq_	
Cl1	−0.06881 (6)	1.08244 (7)	0.09288 (3)	0.0623 (2)	
O1	0.50713 (13)	1.30415 (10)	0.28910 (6)	0.0326 (3)	
N1	0.45035 (14)	0.91350 (11)	0.39374 (6)	0.0244 (3)	
N2	0.26036 (17)	0.87555 (14)	0.46421 (7)	0.0396 (4)	
N3	−0.00227 (17)	1.12750 (16)	0.44726 (8)	0.0423 (5)	
C1	0.56260 (16)	1.20335 (12)	0.30796 (7)	0.0232 (3)	
C2	0.72897 (16)	1.17417 (14)	0.30258 (8)	0.0276 (4)	
C3	0.75310 (17)	1.03530 (14)	0.29019 (8)	0.0291 (4)	
C4	0.69031 (16)	0.95720 (14)	0.34366 (8)	0.0282 (4)	
C5	0.53218 (15)	0.99782 (12)	0.35801 (6)	0.0213 (3)	
C6	0.47011 (15)	1.10911 (12)	0.33829 (6)	0.0206 (3)	
C7	0.30491 (15)	1.14205 (12)	0.34566 (6)	0.0212 (3)	
C8	0.24453 (15)	1.06023 (13)	0.39764 (6)	0.0221 (3)	
C9	0.31330 (16)	0.95091 (13)	0.41841 (7)	0.0236 (3)	
C10	0.50999 (16)	0.78952 (12)	0.40891 (7)	0.0223 (3)	
C11	0.61730 (19)	0.77207 (14)	0.45979 (8)	0.0315 (4)	
C12	0.67482 (19)	0.65278 (16)	0.47317 (8)	0.0340 (4)	
C13	0.62493 (17)	0.54973 (14)	0.43663 (7)	0.0268 (4)	
C14	0.51702 (19)	0.56892 (14)	0.38571 (7)	0.0296 (4)	
C15	0.45943 (17)	0.68829 (14)	0.37163 (7)	0.0270 (4)	
C16	0.6846 (2)	0.42010 (16)	0.45269 (8)	0.0369 (5)	
C17	0.10810 (17)	1.09671 (14)	0.42500 (7)	0.0267 (4)	
C18	0.21047 (15)	1.12918 (13)	0.28167 (7)	0.0215 (3)	
C19	0.12277 (17)	1.22793 (14)	0.25608 (8)	0.0290 (4)	
C20	0.03681 (18)	1.21482 (16)	0.19772 (8)	0.0351 (4)	
C21	0.03801 (18)	1.10148 (18)	0.16578 (8)	0.0341 (5)	
C22	0.12380 (19)	1.00150 (17)	0.19015 (8)	0.0334 (4)	
C23	0.21025 (17)	1.01654 (14)	0.24800 (7)	0.0284 (4)	
H1N	0.31260	0.80530	0.47550	0.0480*	
H2A	0.78620	1.19830	0.34220	0.0330*	
H2B	0.76720	1.22270	0.26760	0.0330*	
H2N	0.17130	0.89510	0.48190	0.0480*	
H3A	0.86130	1.01850	0.28810	0.0350*	
H3B	0.70190	1.01210	0.24910	0.0350*	
H4A	0.68820	0.86940	0.33100	0.0340*	
H4B	0.75760	0.96510	0.38240	0.0340*	
H7	0.29660	1.23540	0.36000	0.0250*	
H11	0.65090	0.84010	0.48500	0.0380*	
H12	0.74800	0.64170	0.50720	0.0410*	
H14	0.48270	0.50090	0.36060	0.0350*	
H15	0.38730	0.70000	0.33730	0.0320*	
H16A	0.63750	0.36050	0.42290	0.0550*	
H16B	0.66120	0.39850	0.49580	0.0550*	
H16C	0.79330	0.41860	0.44960	0.0550*	
H19	0.12140	1.30390	0.27820	0.0350*	
H20	−0.02070	1.28170	0.18050	0.0420*	
H22	0.12370	0.92540	0.16820	0.0400*	
H23	0.26920	0.94990	0.26450	0.0340*	

### Atomic displacement parameters (Å^2^)

**Table:**

	*U*^11^	*U*^22^	*U*^33^	*U*^12^	*U*^13^	*U*^23^
Cl1	0.0483 (3)	0.0933 (5)	0.0424 (3)	0.0171 (3)	−0.0178 (2)	−0.0039 (3)
O1	0.0312 (6)	0.0215 (5)	0.0457 (6)	−0.0005 (4)	0.0073 (5)	0.0058 (4)
N1	0.0234 (6)	0.0192 (5)	0.0318 (6)	0.0035 (4)	0.0106 (4)	0.0033 (4)
N2	0.0389 (8)	0.0354 (7)	0.0476 (8)	0.0117 (6)	0.0251 (6)	0.0171 (6)
N3	0.0319 (7)	0.0531 (9)	0.0439 (8)	0.0113 (7)	0.0168 (6)	0.0056 (7)
C1	0.0228 (6)	0.0203 (6)	0.0267 (6)	−0.0028 (5)	0.0036 (5)	−0.0022 (5)
C2	0.0213 (6)	0.0253 (6)	0.0370 (8)	−0.0037 (5)	0.0069 (5)	0.0027 (6)
C3	0.0231 (7)	0.0277 (7)	0.0377 (8)	0.0004 (5)	0.0121 (6)	0.0016 (6)
C4	0.0222 (6)	0.0250 (6)	0.0383 (8)	0.0032 (5)	0.0095 (5)	0.0040 (6)
C5	0.0206 (6)	0.0200 (6)	0.0238 (6)	−0.0014 (5)	0.0058 (5)	−0.0011 (5)
C6	0.0190 (6)	0.0192 (6)	0.0242 (6)	−0.0001 (4)	0.0050 (4)	−0.0012 (5)
C7	0.0199 (6)	0.0179 (5)	0.0264 (6)	0.0012 (5)	0.0068 (5)	−0.0016 (5)
C8	0.0210 (6)	0.0237 (6)	0.0224 (6)	0.0007 (5)	0.0065 (5)	−0.0017 (5)
C9	0.0229 (6)	0.0235 (6)	0.0253 (6)	0.0004 (5)	0.0084 (5)	0.0001 (5)
C10	0.0235 (6)	0.0197 (6)	0.0242 (6)	0.0023 (5)	0.0063 (5)	0.0025 (5)
C11	0.0362 (8)	0.0255 (7)	0.0319 (7)	0.0001 (6)	−0.0049 (6)	−0.0045 (6)
C12	0.0364 (8)	0.0326 (8)	0.0315 (7)	0.0062 (6)	−0.0081 (6)	0.0014 (6)
C13	0.0302 (7)	0.0257 (6)	0.0249 (6)	0.0060 (5)	0.0054 (5)	0.0042 (5)
C14	0.0371 (8)	0.0230 (6)	0.0282 (7)	0.0024 (6)	−0.0007 (6)	−0.0032 (5)
C15	0.0296 (7)	0.0259 (7)	0.0250 (6)	0.0034 (5)	−0.0016 (5)	−0.0003 (5)
C16	0.0449 (10)	0.0304 (8)	0.0357 (8)	0.0127 (7)	0.0049 (7)	0.0060 (6)
C17	0.0242 (7)	0.0304 (7)	0.0260 (6)	0.0031 (5)	0.0054 (5)	0.0021 (5)
C18	0.0184 (6)	0.0213 (6)	0.0255 (6)	0.0017 (5)	0.0063 (5)	0.0028 (5)
C19	0.0257 (7)	0.0232 (6)	0.0385 (8)	0.0037 (5)	0.0059 (6)	0.0060 (6)
C20	0.0265 (7)	0.0384 (8)	0.0406 (8)	0.0081 (6)	0.0030 (6)	0.0150 (7)
C21	0.0234 (7)	0.0500 (10)	0.0287 (7)	0.0041 (7)	0.0007 (5)	0.0050 (7)
C22	0.0323 (8)	0.0378 (8)	0.0298 (7)	0.0057 (6)	0.0006 (6)	−0.0057 (6)
C23	0.0307 (7)	0.0267 (7)	0.0277 (7)	0.0074 (6)	0.0004 (5)	−0.0005 (5)

### Geometric parameters (Å, º)

**Table:**

Cl1—C21	1.7399 (18)	C14—C15	1.391 (2)
O1—C1	1.2310 (17)	C18—C23	1.388 (2)
N1—C5	1.3943 (18)	C18—C19	1.387 (2)
N1—C9	1.3967 (19)	C19—C20	1.392 (2)
N1—C10	1.4462 (18)	C20—C21	1.377 (3)
N2—C9	1.351 (2)	C21—C22	1.380 (3)
N3—C17	1.149 (2)	C22—C23	1.388 (2)
C1—C2	1.504 (2)	C2—H2A	0.9700
C1—C6	1.4596 (19)	C2—H2B	0.9700
C2—C3	1.517 (2)	C3—H3A	0.9700
N2—H2N	0.9100	C3—H3B	0.9700
N2—H1N	0.9000	C4—H4A	0.9700
C3—C4	1.521 (2)	C4—H4B	0.9700
C4—C5	1.5024 (19)	C7—H7	1.0400
C5—C6	1.3554 (18)	C11—H11	0.9300
C6—C7	1.5093 (19)	C12—H12	0.9300
C7—C18	1.5259 (19)	C14—H14	0.9300
C7—C8	1.5112 (18)	C15—H15	0.9300
C8—C17	1.414 (2)	C16—H16A	0.9600
C8—C9	1.3670 (19)	C16—H16B	0.9600
C10—C15	1.383 (2)	C16—H16C	0.9600
C10—C11	1.381 (2)	C19—H19	0.9300
C11—C12	1.387 (2)	C20—H20	0.9300
C12—C13	1.388 (2)	C22—H22	0.9300
C13—C16	1.505 (2)	C23—H23	0.9300
C13—C14	1.388 (2)		
			
C5—N1—C9	119.95 (11)	Cl1—C21—C20	120.07 (14)
C5—N1—C10	120.75 (12)	C20—C21—C22	121.31 (15)
C9—N1—C10	119.20 (12)	C21—C22—C23	118.85 (16)
O1—C1—C2	121.42 (13)	C18—C23—C22	121.27 (14)
O1—C1—C6	121.11 (13)	C1—C2—H2A	109.00
C2—C1—C6	117.45 (12)	C1—C2—H2B	109.00
C1—C2—C3	111.15 (12)	C3—C2—H2A	109.00
C9—N2—H1N	119.00	C3—C2—H2B	109.00
C9—N2—H2N	120.00	H2A—C2—H2B	108.00
H1N—N2—H2N	121.00	C2—C3—H3A	110.00
C2—C3—C4	110.27 (13)	C2—C3—H3B	110.00
C3—C4—C5	111.89 (12)	C4—C3—H3A	110.00
N1—C5—C6	120.83 (12)	C4—C3—H3B	110.00
N1—C5—C4	115.87 (12)	H3A—C3—H3B	108.00
C4—C5—C6	123.31 (12)	C3—C4—H4A	109.00
C1—C6—C5	120.47 (12)	C3—C4—H4B	109.00
C1—C6—C7	116.75 (11)	C5—C4—H4A	109.00
C5—C6—C7	122.78 (12)	C5—C4—H4B	109.00
C6—C7—C18	111.10 (10)	H4A—C4—H4B	108.00
C8—C7—C18	112.08 (11)	C6—C7—H7	110.00
C6—C7—C8	108.91 (11)	C8—C7—H7	108.00
C7—C8—C17	118.46 (12)	C18—C7—H7	107.00
C7—C8—C9	123.20 (12)	C10—C11—H11	120.00
C9—C8—C17	118.31 (13)	C12—C11—H11	120.00
N1—C9—N2	115.79 (13)	C11—C12—H12	119.00
N1—C9—C8	119.95 (13)	C13—C12—H12	119.00
N2—C9—C8	124.22 (13)	C13—C14—H14	119.00
C11—C10—C15	120.10 (13)	C15—C14—H14	120.00
N1—C10—C11	120.53 (12)	C10—C15—H15	120.00
N1—C10—C15	119.37 (13)	C14—C15—H15	120.00
C10—C11—C12	119.80 (14)	C13—C16—H16A	109.00
C11—C12—C13	121.05 (15)	C13—C16—H16B	109.00
C12—C13—C14	118.40 (14)	C13—C16—H16C	109.00
C14—C13—C16	120.92 (14)	H16A—C16—H16B	110.00
C12—C13—C16	120.68 (14)	H16A—C16—H16C	109.00
C13—C14—C15	120.99 (14)	H16B—C16—H16C	109.00
C10—C15—C14	119.66 (13)	C18—C19—H19	120.00
N3—C17—C8	179.36 (17)	C20—C19—H19	120.00
C7—C18—C19	121.43 (13)	C19—C20—H20	120.00
C7—C18—C23	119.98 (12)	C21—C20—H20	120.00
C19—C18—C23	118.59 (14)	C21—C22—H22	121.00
C18—C19—C20	120.84 (14)	C23—C22—H22	121.00
C19—C20—C21	119.14 (15)	C18—C23—H23	119.00
Cl1—C21—C22	118.62 (14)	C22—C23—H23	119.00
			
C5—N1—C10—C11	81.13 (18)	C6—C7—C8—C17	162.97 (12)
C9—N1—C10—C11	−95.26 (17)	C18—C7—C8—C9	104.62 (15)
C5—N1—C10—C15	−98.38 (16)	C18—C7—C8—C17	−73.69 (15)
C5—N1—C9—N2	−165.14 (13)	C6—C7—C18—C23	55.07 (16)
C10—N1—C9—N2	11.28 (19)	C8—C7—C18—C19	112.49 (15)
C5—N1—C9—C8	12.5 (2)	C8—C7—C18—C23	−67.03 (16)
C10—N1—C9—C8	−171.05 (13)	C6—C7—C18—C19	−125.41 (14)
C9—N1—C5—C4	169.55 (13)	C7—C8—C9—N1	3.4 (2)
C10—N1—C5—C4	−6.82 (18)	C17—C8—C9—N1	−178.31 (13)
C9—N1—C5—C6	−10.23 (19)	C17—C8—C9—N2	−0.8 (2)
C10—N1—C5—C6	173.41 (12)	C7—C8—C9—N2	−179.15 (13)
C9—N1—C10—C15	85.23 (17)	N1—C10—C11—C12	−179.16 (14)
C6—C1—C2—C3	35.47 (18)	C15—C10—C11—C12	0.4 (2)
O1—C1—C6—C5	179.08 (13)	N1—C10—C15—C14	179.60 (13)
C2—C1—C6—C7	177.66 (12)	C11—C10—C15—C14	0.1 (2)
O1—C1—C2—C3	−146.41 (15)	C10—C11—C12—C13	−0.8 (2)
C2—C1—C6—C5	−2.80 (19)	C11—C12—C13—C14	0.7 (2)
O1—C1—C6—C7	−0.5 (2)	C11—C12—C13—C16	−178.22 (15)
C1—C2—C3—C4	−57.68 (17)	C12—C13—C14—C15	−0.3 (2)
C2—C3—C4—C5	47.83 (16)	C16—C13—C14—C15	178.66 (14)
C3—C4—C5—C6	−16.04 (19)	C13—C14—C15—C10	−0.1 (2)
C3—C4—C5—N1	164.19 (12)	C7—C18—C19—C20	−179.83 (14)
N1—C5—C6—C1	172.36 (12)	C23—C18—C19—C20	−0.3 (2)
N1—C5—C6—C7	−8.13 (19)	C7—C18—C23—C22	179.08 (14)
C4—C5—C6—C7	172.11 (12)	C19—C18—C23—C22	−0.5 (2)
C4—C5—C6—C1	−7.4 (2)	C18—C19—C20—C21	0.9 (2)
C1—C6—C7—C8	−159.39 (11)	C19—C20—C21—Cl1	179.82 (13)
C1—C6—C7—C18	76.69 (14)	C19—C20—C21—C22	−0.7 (2)
C5—C6—C7—C8	21.09 (17)	Cl1—C21—C22—C23	179.45 (12)
C5—C6—C7—C18	−102.84 (14)	C20—C21—C22—C23	−0.1 (2)
C6—C7—C8—C9	−18.72 (17)	C21—C22—C23—C18	0.6 (2)

### Hydrogen-bond geometry (Å, º)

Cg3 and Cg4 are the centroids of the methyl- and chloro-benzene rings (C10–C15 and C18–C23), respectively.

**Table:**

*D*—H···*A*	*D*—H	H···*A*	*D*···*A*	*D*—H···*A*
N2—H2*N*···N3^i^	0.91	2.17	3.013 (2)	153
C14—H14···O1^ii^	0.93	2.58	3.4577 (19)	156
C3—H3*A*···*Cg*4^iii^	0.97	2.93	3.7210 (16)	139
C16—H16*B*···*Cg*3^iv^	0.96	2.81	3.6464 (18)	146

Symmetry codes: (i) −*x*, −*y*+2, −*z*+1; (ii) *x*, *y*−1, *z*; (iii) *x*+1, *y*, *z*; (iv) −*x*+1, −*y*+1, −*z*+1.
