# Supplementary figures and images for: Crystal structure of 2-amino-4-(4-chloro­phen­yl)-1-(4-methyl­phen­yl)-5-oxo-1,4,5,6,7,8-hexa­hydro­quinoline-3-carbo­nitrile
Source: Acta Crystallogr E Crystallogr Commun. 2015 Nov 14;71(Pt 12):o949–50. doi: 10.1107/S2056989015021313 (PMC4719917; doi:10.1107/S2056989015021313)

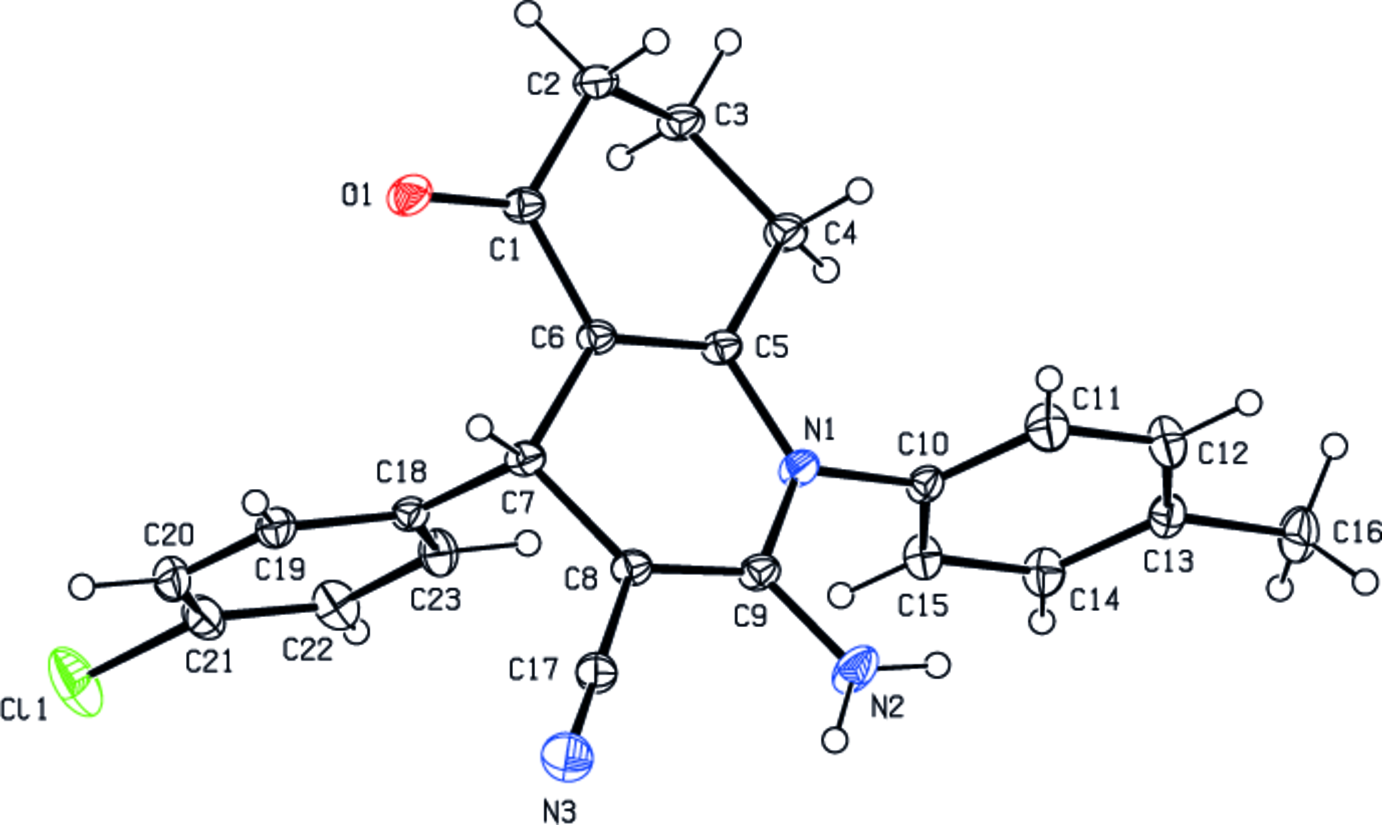

Supplement: Supplementary file 4 [file e-71-0o949-fig1.tif]

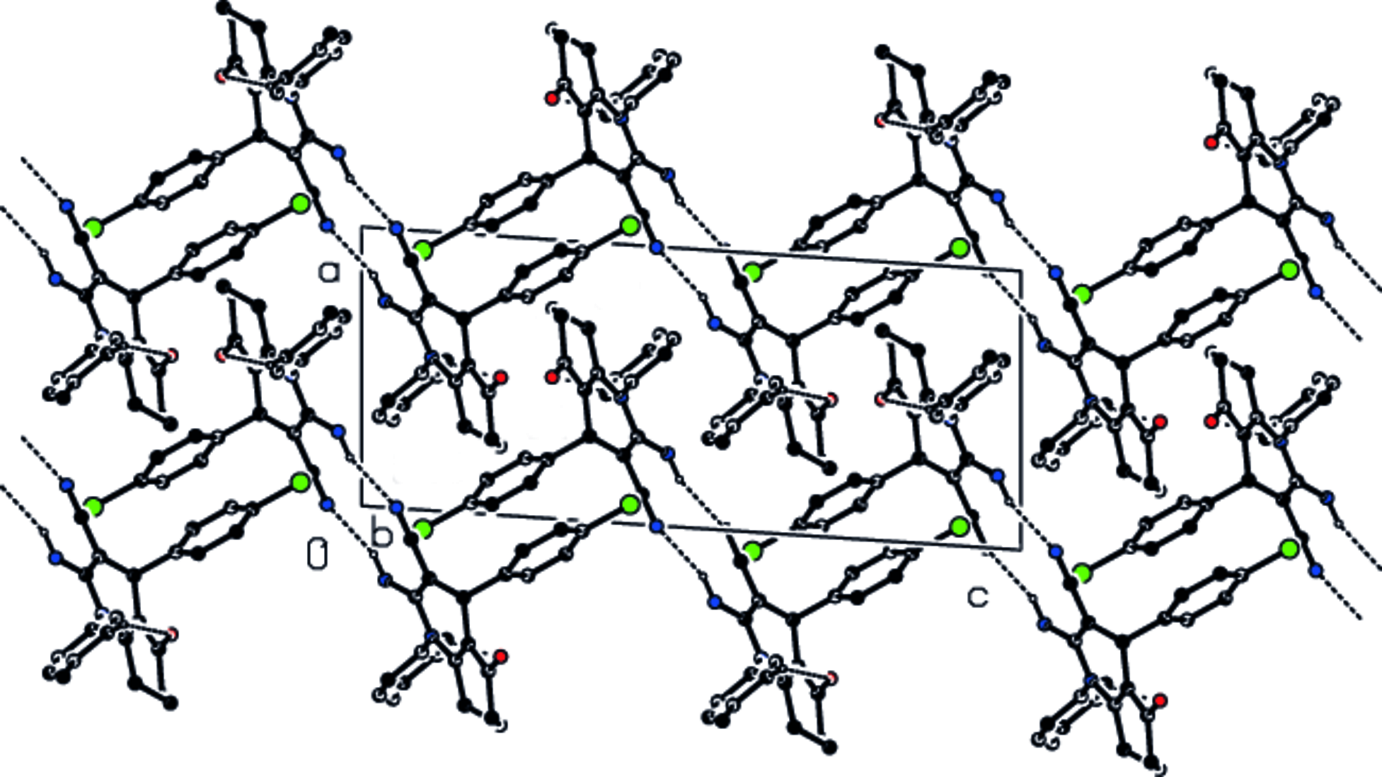

Supplement: Supplementary file 5 [file e-71-0o949-fig2.tif]
